# Supplementary material for: Interventions combining mindfulness training with non-invasive brain stimulation and their impact on mental health outcomes: Protocol for a systematic review and meta-analysis of randomized controlled trials
Source: PLoS One. 2023 Nov 28;18(11):e0288692. doi: 10.1371/journal.pone.0288692 (PMC10684008; doi:10.1371/journal.pone.0288692)
Supplement: S1 Fig — (DOCX) [file pone.0288692.s002.docx]

**Figure 1. Decision strategy for trials independent screening**

Review, book chapter

Humans

NIBS + Mindfulness combination

RCT

Inclusion

Wrong publication type

Wrong population

Wrong drug

Wrong study design

NO

NO

NO

NO

YES

YES

YES

YES
