# Supplementary material for: Interventions combining mindfulness training with non-invasive brain stimulation and their impact on mental health outcomes: Protocol for a systematic review and meta-analysis of randomized controlled trials
Source: PLoS One. 2023 Nov 28;18(11):e0288692. doi: 10.1371/journal.pone.0288692 (PMC10684008; doi:10.1371/journal.pone.0288692)
Supplement: S1 File — (DOCX) [file pone.0288692.s003.docx]

**Document 1. Search strategy for Pubmed**

((transcranial magnetic stimulation[MeSH Terms]) OR ("transcranial magnetic stimulation") OR (rTMS) OR (TMS) OR ("theta burst stimulation") OR (iTBS) OR (dTMS) OR (Transcranial Direct Current Stimulation[MeSH Terms]) OR ("direct current stimulation") OR ("Transcranial Direct Current Stimulation") OR (tDCS) OR ("Cathodal Stimulation") OR ("Anodal Stimulation") OR ("Transcranial Electrical Stimulations") OR ("Repetitive Transcranial Electrical Stimulation") OR ("cranial electrotherapy stimulation") OR (CES) OR ("transcranial random noise stimulation") OR (tRNS) OR ("reduced impedance non‐invasive cortical electrostimulation") OR (RINCE) OR ("non-invasive electrical brain stimulation") OR ("brain stimulation") OR (electrostimulation) OR ("electrostimulation therapy") OR ("non-invasive brain stimulation") OR (NIBS)) AND ((Mindfulness [MeSH Terms]) OR (Mindfulness) OR (Mindfulness-based) OR (MBI) OR (MBSR) OR (MBCT) OR (MBRP) OR (Mindful) OR (meditation))
