# Supplementary material for: Interventions combining mindfulness training with non-invasive brain stimulation and their impact on mental health outcomes: Protocol for a systematic review and meta-analysis of randomized controlled trials
Source: PLoS One. 2023 Nov 28;18(11):e0288692. doi: 10.1371/journal.pone.0288692 (PMC10684008; doi:10.1371/journal.pone.0288692)
Supplement: S2 File — (DOCX) [file pone.0288692.s004.docx]

| **First author, year** | **Country** | **Design** | **Condition or healthy, mindfulness experience** | **Population (N, % female), mean age** | **Intervention (type, characteristics)** | **Control (characteristics)** | **Timeline** | **Outcomes and scales** | **Results (interpretation)** |
| --- | --- | --- | --- | --- | --- | --- | --- | --- | --- |
|  |  |  |  |  |  |  |  |  |  |
|  |  |  |  |  |  |  |  |  |  |
|  |  |  |  |  |  |  |  |  |  |
|  |  |  |  |  |  |  |  |  |  |
|  |  |  |  |  |  |  |  |  |  |

**Document 2. Extraction form**
