# Supplementary material for: Interventions combining mindfulness training with non-invasive brain stimulation and their impact on mental health outcomes: Protocol for a systematic review and meta-analysis of randomized controlled trials
Source: PLoS One. 2023 Nov 28;18(11):e0288692. doi: 10.1371/journal.pone.0288692 (PMC10684008; doi:10.1371/journal.pone.0288692)
Supplement: S3 File — (DOCX) [file pone.0288692.s005.docx]

| Author, year | Overall N = | Intervention N = | Control N = | Outcome(s) | Timepoints | Mean (SD) int baseline | Mean (SD) int | Mean (SD) control baseline | Mean (SD) control |
| --- | --- | --- | --- | --- | --- | --- | --- | --- | --- |
|  |  |  |  |  |  |  |  |  |  |
|  |  |  |  |  |  |  |  |  |  |
|  |  |  |  |  |  |  |  |  |  |
|  |  |  |  |  |  |  |  |  |  |
|  |  |  |  |  |  |  |  |  |  |

**Document 3. Results, numerical values for each included outcome**
