# Supplementary material for: Interventions combining mindfulness training with non-invasive brain stimulation and their impact on mental health outcomes: Protocol for a systematic review and meta-analysis of randomized controlled trials
Source: PLoS One. 2023 Nov 28;18(11):e0288692. doi: 10.1371/journal.pone.0288692 (PMC10684008; doi:10.1371/journal.pone.0288692)
Supplement: Attachment — Submitted filename: Response to reviewers.docx [file pone.0288692.s006.docx]

Université de Bourgogne

7 Boulevard Jeanne d'Arc

21000 Dijon France

[anastasia.demina@chu-dijon.fr](mailto:anastasia.demina@chu-dijon.fr)

**PLOS ONE**

June 20^th^, 2023

Dear reviewers,

We wish to thank you for your generous comments on our paper.

We wish to submit the requested minor revisions to our systematic review and meta-analysis protocol entitled **Interventions combining mindfulness training with non-invasive brain stimulation and their impact on mental health outcomes: protocol for a systematic review and meta-analysis of randomized controlled trials.**

We addressed all concerns about PLOS ONE's style requirements and we added captions for our Supporting Information files. We confirm that we did not cite any retracted paper.

We also edited our manuscript to address all the concerns pointed out by our reviewers.

**First reviewer’s concerns:**

- We specified the guarantor of the study (L417)
- We added requested details to our Abstract (eligibility criteria and sources as well as software details) (L26-28, L31-33)
- We merged Outcomes and Outcomes and Prioritization sections as requested (L130-177)
- The post-COVID mental health status explanation and WHO definition of mental health were removed.
- We insisted on the separation of clinical and lab studies analyses in our final review (L113-114)
- In our final review we will discuss the issues of generalizability of the findings related to the study settings, among other factors.
- We planned different subgroup analyses in parallel with a pooled pairwise meta-analysis (L287-290)
- We provided different endpoints for a more comprehensive and nuanced analysis of our data (L263-265)
- We provided our strategy in case of multiple psychometric scales for a single outcome (L274-276)
- We added strategy for handling potential conflicts of interest (L215-216), results will be discussed considering this information.
- All requested changes (L49, L69, L91, L197-201, 206-208, 220-221) were made.

**Second reviewer’s concerns:**

- Rationale and objectives sections were separated as requested (L94-95)
- We merged Outcomes and Outcomes and Prioritization sections as requested (L130-177)
- We added requested references to support statements pointed out by the second reviewer (L79-80)
- We added our strategy to achieve consensus on studies included in pooled pairwise meta-analysis (L274-276)

Please address all correspondence concerning this manuscript to me at anastasia.demina@chu-dijon.fr.

Thank you for your consideration of our work.

Sincerely,

Dr Anastasia DEMINA, M.D., Ph.D. student, corresponding author

Addiction medicine, University of Burgundy, Dijon University Hospital

Each author agrees with the changes made to the manuscript.

**Dr Anastasia Demina, M.D., Ph.D. student**

University of Burgundy, Dijon University Hospital

anastasia.demina@chu-dijon.fr

**Dr Benjamin Petit, M.D.**

University of Burgundy, Dijon University Hospital

benjamin.petit@chu-dijon.fr

**Dr Vincent Meille, M.D.**

University of Burgundy, Dijon University Hospital

vincent.meille@chu-dijon.fr

**Florent Lebon, Ph.D.**

University of Burgundy

florent.lebon@u-bourgogne.fr

**Professor Benoit Trojak, M.D., Ph.D.**

University of Burgundy, Dijon University Hospital

benoit.trojak@chu-dijon.

AUTHOR INFORMATION: AD is an addiction physician and a Ph.D. student at INSERM U1093, CAPS laboratory at the University of Burgundy. BT is her thesis director and FL is her co-director. AD, BT, BP and VM are addiction physicians with experience in designing and curating systematic reviews. The systematic review planned in this protocol will be a part of AD’s Ph.D. dissertation. This systematic review will enable the research team to plan an original experimental research project on MBI combined with NIBS in addiction patients.
